# Association between microRNA 21 expression in serum and lung cancer: A protocol of systematic review and meta-analysis

**DOI:** 10.1097/MD.0000000000020314

**Published:** 2020-05-29

**Authors:** Feng Qiao, Peng Luo, Chun-hui Liu, Kai Fu, Yan-bin Zhao

**Affiliations:** a Department of Cardiothoracic Surgery; b Department of Health Management Medical Examination Center, First Affiliated Hospital of Jiamusi University, Jiamusi; c Department of Chest Surgery, First Affiliated Hospital of Harbin Medical University; d Fifth Ward of Internal Medicine Department, Affiliated Cancer Hospital of Harbin Medical University, Harbin, China.

**Keywords:** association, case-controlled study, lung cancer, microRNA 21

## Abstract

**Background:**

Previous studies have reported that microRNA 21 (mRNA 21) has involved in the procedure of lung cancer (LC). However, its conclusions are still unclear. Thus, this study will try to elaborate the association between mRNA 21 expression in serum and LC.

**Methods:**

The electronic databases of Cochrane Library, PubMed, EMBASE, Allied and Complementary Medicine Database, WANGFANG database, and China National Knowledge Infrastructure will be retrieved from the inception to the present. All electronic databases will be searched without limitations of language and geographical location. Case-controlled studies reporting the association between mRNA 21 expression in serum and LC will be included. In addition, we will also identify other literature sources to avoid missing potential studies. All study selection, information collection, and study quality assessment will be performed by 2 independent authors. RevMan V.5.3 software and Stata V.12.0 software will be used for data synthesis and analysis.

**Results:**

This study will summarize current evidence to investigate the association between mRNA 21 expression in serum and LC.

**Conclusion:**

The findings of this study will present comprehensive evidence to determine whether mRNA 21 expression in serum is relevant with LC or not.

**Systematic review registration:**

INPLASY202040055.

## 1 Introduction

Lung cancer (LC) is one of the most malignant and killing cancers in humans, which is also the leading cause of cancer death.^[1–5]^ Studies have reported that about 116,300 men and 112,520 women will be diagnosed with LC, and about 72,500 men and 63,220 women will die from LC in 2020.^[6]^ Other studies have reported that there are 8.2 million patients with LC died annually till to the present.^[4]^ It has been estimated that this number will rise to 10 million each year by 2030.^[7]^ Despite there are a range of interventions available for LC, all their efficacy is still limited.^[8–15]^ Therefore, it is very important to diagnose LC at early stage.

Previous studies have reported that biomarkers can help to diagnose LC at early stage, such as that microRNA 21 (mRNA 21).^[16–30]^ However, there is no evidence clue of this systematic review and meta-analysis focusing on the association between mRNA 21 expression in serum and LC. Thus, the aim of this study is to investigate the association between mRNA 21 expression in serum and LC.

## 2 Methods and design

### 2.1 Study registration

The protocol has been registered on INPLASY202040055. This study has been reported according to the guidelines of the Cochrane Handbook for Systematic Reviews of Interventions and the Preferred Reporting Items for Systematic Reviews and Meta-Analyses statement.^[31]^

### 2.2 Inclusion criteria for study selection

#### 2.2.1 Type of studies

All case-controlled studies reporting association between mRNA 21 expression in serum and patients with LC will be included. Case reports, case series, reviews, and any other studies will be excluded.

#### 2.2.2 Type of participants

Patients diagnosed as histopathology-proven LC or normal participants will be included in this study. We will not apply any restrictions related to the race, age, and sex.

#### 2.2.3 Type of index test

Experimental group: All patients with histopathology-proven LC examined with the levels of mRNA 21 expression in serum.

Control group: All normal participants were detected with the levels of mRNA 21 expression in serum.

#### 2.2.4 Type of outcome measurements

The primary outcome is levels of serum mRNA-21 (as measured by real-time quantitative real-time polymerase chain reaction).

The secondary outcomes are pathological types, tumor-node-metastasis stages, lymph node metastasis, and tumor markers.

### 2.3 Data sources and search strategy

#### 2.3.1 Electronic searches

We will search Cochrane Library, PubMed, EMBASE, Allied and Complementary Medicine Database, WANGFANG database, and China National Knowledge Infrastructure from the inception to the present. We will search all these electronic databases with no language and geographical location restrictions. We will create detailed search strategy of PubMed in Table 1. We will also adapt similar search strategies of other electronic databases.

**Table 1:**
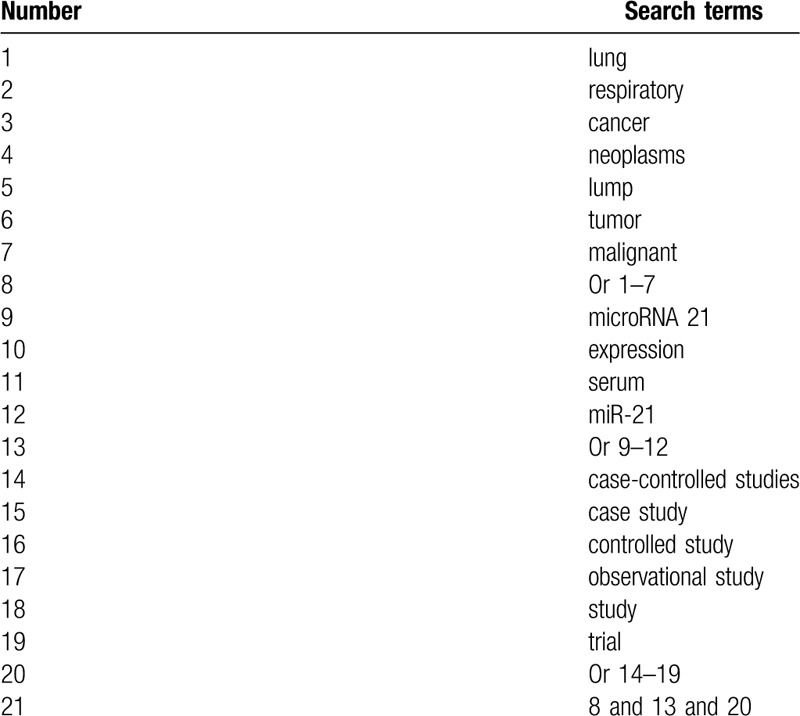
Search strategy for PubMed.

#### 2.3.2 Other resources

Besides the electronic databases, we will search conference proceedings, dissertations, and reference lists of included studies.

### 2.4 Study selection and data collection

#### 2.4.1 Study selection

Two authors will independently examine the titles/abstracts of all identified literatures, and all unrelated studies will be excluded. Then, full-text of potential relevant papers will be obtained to judge whether they fulfill all inclusion criteria. Any discrepancies will be solved by consensus with the help of another experienced author. All excluded studies with detailed reasons will be recorded at different stages. The flowchart of study selection will be presented with details.

#### 2.4.2 Data extraction

Two authors will independently collect the information from each included study. Any conflicts between them will be solved by consensus with the help of a third experienced author. The extracted information consists of manuscript title, name of first author, journal, year of publication, country, race, age, sex, eligibility criteria, sample size, study setting, study design, types of indexes, and outcome indicators. We will contact primary authors to obtain any missing or unclear information from the included studies.

### 2.5 Study quality assessment

In this study, 2 authors will independently assess the study quality for all included studies. Any different opinions will be solved by another experienced author through discussion. We will utilize Newcastle-Ottawa Scale to investigate the methodological quality for observational studies.^[32]^

### 2.6 Assessment of heterogeneity

We will examine the heterogeneity across included trials using *I*^2^ statistic. *I*^2^ ≤ 50% means minor heterogeneity, and we will use a fixed-effects model. On the other hand, *I*^2^ > 50% indicates significant heterogeneity, and we will present a random-effects model.

### 2.7 Statistical analysis

#### 2.7.1 Data synthesis

We will employ RevMan V.5.3 software (Cochrane Community; City, London; Country, UK) for statistical analysis. We will calculate continuous data as mean difference or standardized mean difference and 95% confidence intervals (CIs), and dichotomous data using risk ratio and 95% CIs. We will examine heterogeneity across included studies using *I*^2^ test. *I*^2^ ≤ 50% shows homogeneity and a fixed-effect model will be used, and *I*^2^ > 50% suggests remarkable heterogeneity and a random-effect model will be applied. If possible, we will perform a meta-analysis if we collect ample data from eligible trials with homogeneity. If there is obvious heterogeneity, we will test the sources of heterogeneity. We will also carry out a narrative description.

#### 2.7.2 Subgroup analysis

We will perform subgroup analysis to explore the possible sources of significant heterogeneity based on the different types of study information, types of patients, and outcomes.

#### 2.7.3 Sensitivity analysis

In case of the sufficient data, sensitivity analysis will be performed to test the robustness of merged results by excluding low quality studies.

#### 2.7.4 Reporting bias

We will undertake funnel plots and associated regression test to investigate the reporting bias when at least 10 studies are included.^[33]^

### 2.8 Ethics and dissemination

This study does not inquire ethic approval as original data will not be collected. Its findings will be disseminated though a peer-reviewed journal or a conference.

## 3 Discussion

LC is tough health problem which is difficult to cure but easy to advance, associated with a variety of biomarkers. Although lots of studies have explored this topic, the association between mRNA 25 expression in serum and LC is still not conclusive. We hypothesized that mRNA 25 expression in serum may have close association with LC, but not all evidence support it. Thus, this study aims to systematically assess the association between mRNA 25 expression in serum and LC. It will provide evidence for future studies.

## Author contributions

**Conceptualization:** Feng Qiao, Peng Luo.

**Data curation:** Feng Qiao, Chun-hui Liu, Kai Fu, Yan-bin Zhao.

**Formal analysis:** Feng Qiao, Peng Luo, Kai Fu, Yan-bin Zhao.

**Funding acquisition:** Feng Qiao.

**Investigation:** Feng Qiao.

**Methodology:** Peng Luo, Chun-hui Liu, Kai Fu, Yan-bin Zhao.

**Project administration:** Feng Qiao.

**Resources:** Peng Luo, Chun-hui Liu, Kai Fu, Yan-bin Zhao.

**Software:** Peng Luo, Chun-hui Liu, Kai Fu, Yan-bin Zhao.

**Supervision:** Feng Qiao.

**Validation:** Feng Qiao, Yan-bin Zhao.

**Visualization:** Feng Qiao, Peng Luo, Chun-hui Liu, Kai Fu, Yan-bin Zhao.

**Writing – original draft:** Feng Qiao, Chun-hui Liu, Yan-bin Zhao.

**Writing – review & editing:** Feng Qiao, Peng Luo, Kai Fu, Yan-bin Zhao.
